# Characterization of Surface Topography Variation in the Ultra-Precision Tool Servo-Based Diamond Cutting of 3D Microstructured Surfaces

**DOI:** 10.3390/mi12121448

**Published:** 2021-11-26

**Authors:** Wei Yuan, Chi-Fai Cheung

**Affiliations:** State Key Laboratory in Ultra-Precision Machining Technology, Department of Industrial and Systems Engineering, The Hong Kong Polytechnic University, Hong Kong 999077, China; wer.yuan@connect.polyu.hk

**Keywords:** dynamic modeling, surface characterization, cutting forces, tool servo diamond cutting, data-dependent systems, surface topography variation, ultra-precision machining, microstructured surfaces, microlens array

## Abstract

Previous models of the relative tool-work vibration are not generalized to represent the surface generation mechanism in the ultra-precision tool servo-based diamond cutting (UTSDC) of three-dimensional (3D) microstructured surfaces. This is due to the fact that the tool-work vibration in UTSDC is no longer a steady harmonic vibration with a constant amplitude but is influenced by the tool motion along the thrust direction. In this paper, dynamic modeling of the cutting system is presented for the characterization of surface topography variation in UTSDC of a microlens array considering the tool-work vibration as an underdamped vibration. The natural frequency and damping ratio of the cutting system are determined by the data-dependent systems (DDS) method. Based on the analysis of the surface profile and cutting force signals, it is found that the tool-work vibration is significantly enhanced in the cut-in process when the cutting speed increases. The simulation results show that the proposed dynamic model can well-determine root-mean-squares RMS values of the surface primary profile and the dynamic force acting on the force sensor. The dynamic model provides insight into the formation of the surface topography variation in UTSDC of 3D microstructured surfaces, and the model might be applied in self-optimized machining systems in the future.

## 1. Introduction

Machining instability is a great obstacle to fine surface quality in ultra-precision machining. There has been a wealth of research on establishing an accurate surface topography model according to the cutting mechanics and the dynamics of the machining process. Surface topography in ultra-precision machining is mainly generated by the interaction of tool profiles with a workpiece. The surface finish is mainly characterized by process parameters, tool geometry, material properties and the vibrations arising in the material removal process [1]. Much work has been conducted to investigate the cutting mechanics at the micro/nanometer scales recently. Venkatachalam et al. [2] presented that the flow stress affected by the material grain size, grain boundary and crystallographic orientation contributes to the cutting forces in face-turning of polycrystalline brittle material. Yan et al. [3] proposed that the unsteadiness of side flow of material in the cutting region causes surface waviness perpendicular to the cutting direction when cutting a microgroove array on an electroless-plated NiP surface. In micro-grooving experiments, Guo et al. [4] found that the loss of harder particles in rapidly solidified aluminum due to a high-stress concentration around the straight cutting edges leads to surface and subsurface damage. Rahman et al. [5,6] investigated the cutting-edge radius effect on the surface generation mechanisms and concluded that when the relative tool sharpness is less than 1, the material removal transfers from shearing to extrusion, ploughing and rubbing. Due to the effects of cutting edge, the workpiece surface improves in 2D vibration-assisted machining (VAM) because ploughing increases significantly in 2D VAM [6].

The analysis of the surface data and cutting force signal provides insightful understanding on the formation of surface topography variation. Kim [7] proposed a 3D surface topography simulation model for face-turning aluminum alloy and copper alloy based on the frequency domain information obtained by the Fast Fourier Transform analysis method. Wang et al. [8] pointed out that the Fast Fourier Transform (FFT) method has no access to the localized details of the surface profile. They employed the wavelet method to evaluate the amplitude and frequency of the surface profile signal along the cutting direction. By applying the data-dependent systems (DDS) approach to analyze the surface profile, Pandit [9] found that the relative tool-work vibration plays a dominant role in the surface generation in ultra-precision diamond turning. Takasu et al. [10] pointed out that the relative tool-work vibration commonly has a small amplitude and low frequency. The surface roughness in the tool feed direction can be much smaller than the amplitude of tool-work vibration by optimizing the phase shift of the vibration to one spindle revolution. Cheung and Lee [11] proposed that the spindle run-out and machine vibration are the major sources of the tool-work vibration in ultra-precision diamond turning. The decomposition of the surface roughness profile indicated that the cutting dynamics of the surface roughness have a strong correlation with the surface generation mechanisms. The natural frequency component which results from the tool-work vibration contributes to more than 67% of the total surface roughness. The feed and tool geometry component contributes to much less percentage of the total roughness as compared to conventional machining [12]. Chen and Zhao [13] pointed out that the actual tool-work vibration during the turning process is different from the measured vibration before turning, and the actual relative vibration is almost impossible to measure directly in the cutting process.

The above-mentioned studies mainly focus on the simple cutting operation, where the depth of the cut does not change in the whole material removal process. The tool-work vibration is assumed to be a steady harmonic vibration with a constant amplitude and frequency, which produces periodic patterns on a machined surface [14], while in the ultra-precision tool servo-based diamond cutting (UTSDC), 3D microstructures such as microlens arrays are cut by the servo-controlled motion of the diamond tool. The force in the thrust direction, which is significantly affected by the cut-in and cut-out process, may cause extra material to be indented beneath the tool. The indentation force and the tool servo motion lead to the instability in machining and result in surface topography variation. Slowing down the cutting velocity is found to be one solution to reduce the surface topography variation [15]. However, there is still a lack of a dynamic model to determine the effect of machine dynamics on the surface topography variation and how it is affected by the cutting speed.

This paper aims to investigate the surface generation mechanism in UTSDC considering the effect of the servo motion in the thrust direction and the thrust force corresponding to the material being indented as the diamond tool cuts into the surface. A dynamic model is proposed for analyzing the surface topography variation in the UTSDC of a microlens array. The model is built by the identification of the dynamic parameters of the system based on the DDS method and the estimation of the contact force in the thrust direction during machining. Hence, the equation of motion of the cutting system is derived to determine the transient response of the diamond tool. The simulation results are presented and validated by a series of cutting experiments and the analysis of the surface profiles of machined surfaces.

## 2. Characterization Method for Surface Topography Variation

**Figure 1:**
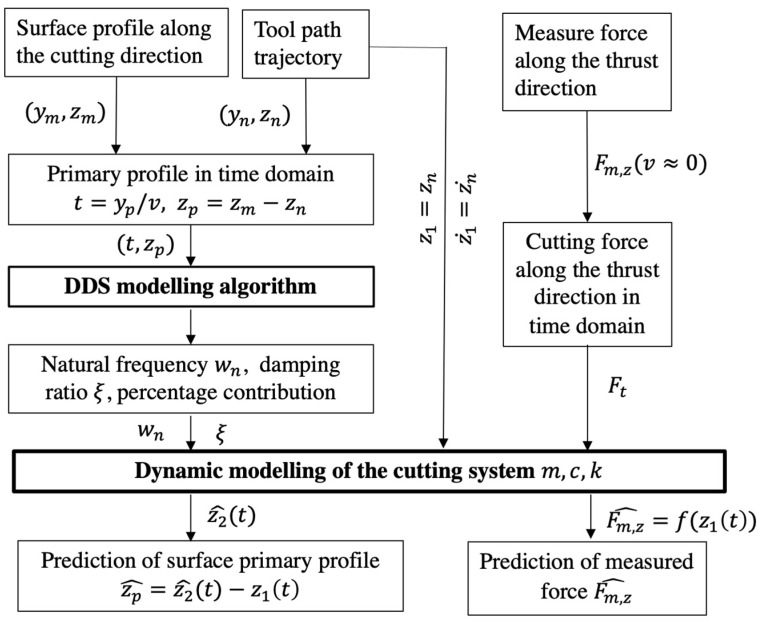
Framework for the characterization of the surface topography variation in ultra-precision tool servo-based diamond cutting (UTSDC).

Figure 1 shows a framework for the characterization of the surface topography variation in the UTSDC of microstructured surfaces. To eliminate the potential spindle vibration, the microstructures are cut only by two slides of the machine tool, i.e., the *y*-axis and *z*-axis. In this case, the surface profile along the cutting direction provides a faithful signature of the machining instability. The primary profile is obtained by subtracting the form from the measured surface profile. The natural frequency and damping ratio of the tool-work vibration are determined by a DDS model which provides component-by-component wavelength decomposition of the surface primary profile of the workpiece. During the machining process, the cutting force along the thrust direction is determined when the cutting speed is low so that the vibration is insignificant. The measured thrust force is used as an input of the dynamic cutting system. Hence, a dynamic model of the cutting system is built. The variation of surface topography and the dynamic force in the condition of higher cutting speed are predicted by solving the dynamic equation.

### 2.1. Theoretical Background of the Data-Dependent Systems Analysis Method

The surface primary profile $\left(y_{p},z_{p}\right)$ of the workpiece is the sum of all the deviations of the measured profile $\left(y_{m},z_{m}\right)$ from the nominal profile. The nominal profile $\left(y_{n},z_{n}\right)$ is assumed to be the vertical projection of the ideal tool path trajectory on the designed surface for UTSDC. Thus,

(1) $$\left(y_{p},z_{p}\right)=\left(\;y_{n},z_{m}-z_{n}\right)\;$$

The measured surface profile is an unmodified profile, which is composed of a wide range of frequency components. For example, the profile that only contains the high-frequency components of the surface profile is denoted as the surface roughness profile. To determine the effect of the tool-work vibration on the surface roughness components or the waviness components, the surface profile is used as raw data for analyzing the natural frequency and damping ratio of the tool-work vibration. 

Data-dependent systems (DDS) is a comprehensive methodology for the modeling and characterization of surfaces, which can relate the surface topography to the manufacturing process [16]. DDS is developed based on the Fundamental Theorem [17] that any stochastic system can be represented by the response of an n-pole–zero transfer function to white noise. Thus, the differential equation for the system response, $X\left(t\right)$, is expressed as:

(2) $$\frac{d^{n}X\left(t\right)}{dt^{n}}+\alpha_{n-1}\frac{d^{n}X\left(t\right)}{dt^{n}}+\ldots +\alpha_{0}X\left(t\right)=b_{n-1}\frac{d^{n-1}Z\left(t\right)}{dt^{n-1}}+\ldots +b_{1}\frac{d^{1}Z\left(t\right)}{dt^{1}}+Z\left(t\right)\;$$

where $Z\left(t\right)$ is white noise, $\alpha_{i}$ is an autoregressive (AR) parameter and $b_{i}$ is a moving average (MA) parameter. Equation (2) is denoted by ARMA$\left(n,n-1\right)$. If some $b_{i}$ are zero, the ARMA$\left(n,n-1\right)$ model can be simplified as an ARMA $\left(n,m\right)$ model, where m≤n−1. According to the Uniform Sampling Theorem theory [17], an ARMA$\left(n,n-1\right)$ system can be represented by the following stochastic difference equation as long as the data are sampled at a uniform interval of ∆:

(3) $$X_{t}-\phi_{1}X_{t-1}-\ldots -\phi_{n}X_{t-n}=a_{t}-\theta_{1}a_{t-1}-\theta_{n-1}a_{t-n+1}$$

With the backstep notation $BX_{t}=X_{t-1}$, the autoregressive part of the ARMA$\left(n,n-1\right)$ model, i.e., the left-hand side of Equation (3), can be expressed in the form of backward shift operator B,

(4) $$\left(1-\phi_{1}B-\phi_{2}B^{2}-\ldots -\phi_{n}B^{n}\right)X_{t}\equiv \left(1-\lambda_{1}B\right)\left(1-\lambda_{2}B\right)\ldots \left(1-\lambda_{n}B\right)X_{t}\;$$

where $\lambda_{i}$ are the roots of the characteristic equation, given by:

(5) $$\lambda^{n}-\phi_{1}\lambda^{n-1}-\phi_{2}\lambda^{n-2}\ldots -\phi_{n}=0\;$$

Similarly, the right-hand side of Equation (3) can be expressed as:

(6) $$a_{t}-\theta_{1}a_{t-1}-\theta_{n-1}a_{t-n+1}\equiv \left(1-\theta_{1}B-\theta_{2}B^{2}-\ldots -\theta_{n}B^{n}\right)a_{t}$$

Substituting Equations (4) and (6) into Equation (3), then:

(7) $$X_{t}=\frac{\left(1-\theta_{1}B-\theta_{2}B^{2}-\ldots -\theta_{n}B^{n}\right)\;}{\left(1-\lambda_{1}B\right)\left(1-\lambda_{2}B\right)\ldots \left(1-\lambda_{n}B\right)}a_{t}$$

Equation (7) can also be expressed by a group of Green’s functions, $G_{j}$:

(8) $$X_{t}=\overset{\infty }{\underset{j=0}{\sum }}G_{j}a_{t-j}$$

(9) $$G_{j}=g_{1}\lambda_{1}^{j}+g_{2}\lambda_{2}^{j}+\ldots +g_{n}\lambda_{n}^{j}$$

(10) $$g_{i}=\frac{\lambda_{i}^{n-1}-\theta_{1}\lambda_{i}^{n-2}-\ldots -\theta_{n-1}}{\prod_{j=1,j\neq i}^{n}\left(\lambda_{i}-\lambda_{j}\right)}\;$$

The characteristic root, $\lambda_{i}$, can be either a real number or a complex number. A real $\lambda_{i}$ represents a decaying exponential dynamic mode, while a pair of complex roots, $\lambda_{i}$,$\;\lambda_{i}^{*}$, describes a damped or undamped harmonic vibration mode. The natural frequency, $\omega_{i}$, and damping ratio, $\zeta_{i}$, corresponding to the pair of complex roots, $\lambda_{i}$,$\;\lambda_{i}^{*}$, are obtained as [18]:

(11) $$\omega_{i}=\sqrt{\sigma_{i}^{2}+\Omega_{i}^{2}}\;$$

(12) $$\zeta_{i}=-\frac{\sigma_{i}}{\sqrt{\sigma_{i}^{2}+\Omega_{i}^{2}}\;}$$

where $\sigma_{i}$ and $\Omega_{i}$ are defined by:

(13) $$\sigma_{i}=\frac{1}{2∆}In\left(\lambda_{i}\lambda_{i}^{*}\right)\;$$

(14) $$\Omega_{i}=\frac{1}{∆}\arctan\left[\frac{Im\left(\lambda_{i}\right)}{Re\left(\lambda_{i}\right)}\right]\;$$

The power of a root, $\lambda_{i}$, is expressed by $d_{i}$:

(15) $$d_{i}=\sum_{j=1}^{n}\frac{g_{i}g_{j}}{1-\lambda_{i}\lambda_{j}}$$

If $\lambda_{i}$ is a real root, the percentage contribution of the total power is given by:

(16) $$P=\frac{d_{i}}{\sum_{j=1}^{n}d_{j}}\times 100\;$$

If $\lambda_{i}$ and $\lambda_{i+1}$ are a pair of complex conjugate roots, the percentage contribution of the power of this vibration mode to the total power is expressed by:

(17) $$P=\frac{d_{i}+d_{i+1}}{\sum_{j=1}^{n}d_{j}}\times 100\;$$

In practice, ARMA$\left(2n,2n-1\right)$ models are used to represent an n-degrees-of-freedom vibration system. The order 2n is found by an iterative, least-squares procedure until the increase in order fails to significantly reduce the residual sum of squares [18]. The significant improvement in fit is checked by the *F*-test:

(18) $$F=\frac{\left(E_{1}-E_{0}\right)/S}{E_{0}/\left(N-r\right)}\sim F\left(S,N-r\right)$$

where N is the number of the input data, $E_{0}$ is the residual sum of squares of the ARM$\left(2n+2,\;2n+1\right)$ model and $E_{1}$ is the residual sum of squares of the ARMA$\left(2n,2n-1\right)$ model. The F distribution has two degrees of freedom, denoted by S and N−r, respectively. The values of S and r are expressed as:

(19) S=4

(20) r=4n+3 

The *F*-test starts with the ARMA$\left(2,1\right)$ and ARMA$\left(4,3\right)$ models. If the F value is less than the value of $F\left(S,N-r\right)$ at a 5% level of significance, then ARMA$\left(2,1\right)$ is adequate to represent the system. Otherwise, ARMA$\left(2,1\right)$ is not sufficient, so an *F*-test comparing ARMA$\left(4,3\right)$ and ARMA$\left(6,5\right)$ would be conducted to check whether ARMA$\left(4,3\right)$ is adequate. The iterative procedure is carried on until the F value does not exceed the value of $F\left(S,N-r\right)$ at a 5% level of significance.

### 2.2. Dynamic Modeling of Relative Tool-Work Vibration 

**Figure 2:**
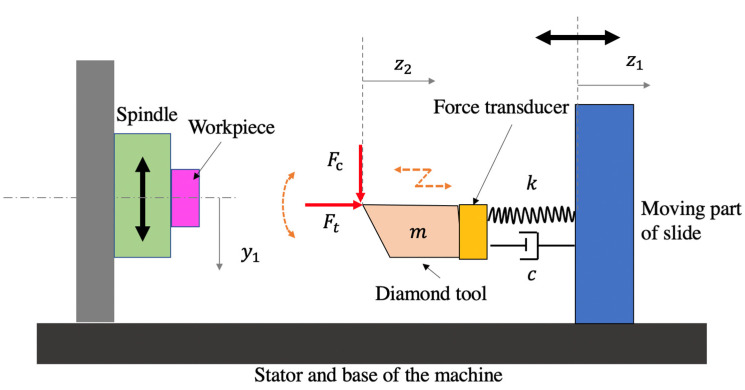
The mechanical structure of the cutting system.

Figure 2 shows the mechanical structure of the cutting system. The workpiece is mounted on the spindle which moves along the *y*-axis during the UTSDC operation. The displacement of the moving part of the slide, the diamond tool and workpiece are $z_{1}$, $z_{2}$ and $y_{1}$, respectively. The moving part of the slide is regarded as a base, which is assumed to precisely follow the commands consisting of the tool path trajectory. A force transducer is placed between the diamond tool system and the moving part of the slide.

The dynamic system is simplified as a single-degree-of-freedom system with a moving base. Based on Newton’s second law, the equation of the motion of the diamond tool system is given by:

(21) $$m{\ddot{z}}_{2}\left(t\right)+c\left({\dot{z}}_{2}\left(t\right)-{\dot{z}}_{1}\left(t\right)\right)+k\left(z_{2}\left(t\right)-z_{1}\left(t\right)\right)=F_{t}\left(t\right)$$

where $z_{1}$(*t*) can be obtained from the commanded tool path. The thrust force, $F_{t}\left(t\right)$, in the *z*-axis direction is not actually the measured force, $F_{m,z}$, obtained by the force sensor. $F_{m,z}$ is the total force applied on the force transducer, which is expressed by:

(22) $$F_{m,z}\left(t\right)=F_{t}\left(t\right)-c\left({\dot{z}}_{2}\left(t\right)-{\dot{z}}_{1}\left(t\right)\right)-k\left(z_{2}\left(t\right)-z_{1}\left(t\right)\right)\;$$

The initial condition $(z_{2}\left(t=0\right)$,$\;\dot{z_{2}}\left(t=0\right))$ is determined by the tool path and operation parameters.

When the cutting speed is extremely low, the system can be regarded as a “semi-static” system, where no obvious vibration signal can be detected. Under such condition, the measured force, $F_{m,z}\left(t\right)$, is close to the thrust force, $F_{t}\left(t\right)$.

(23) $$F_{t}\left(t\right)\approx F_{m,z}\left(t,v\approx 0\right)\;$$

The solution of Equation (21) can be solved by a numerical method. It is noted that $\left(y_{1},z_{1}\right)$ is assumed to be equal to the generated tool path $\left(y_{n},z_{n}\right)$, and $\left(y_{1},z_{2}\right)$ is assumed to be equal to the actual measured profile ($y_{m},z_{m})$. Thus, the simulated primary profile, $z_{p}$, is obtained by:

(24) $$z_{p}=z_{2}-z_{1}=z_{m}-z_{n}$$

## 3. Experimental Setup

**Figure 3:**
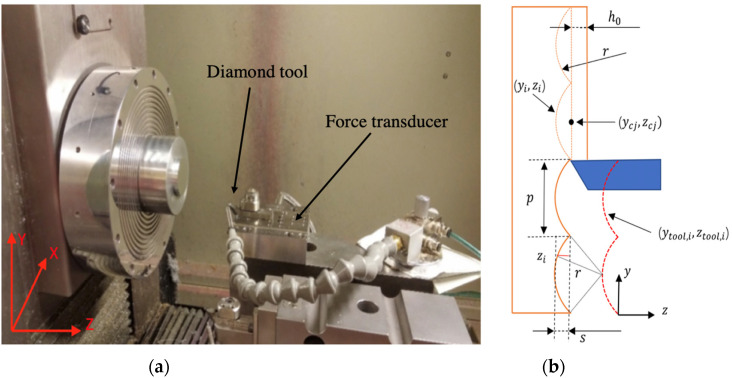
(**a**) Experiment setup. (**b**) Tool path for cutting a microlens array.

To investigate the characteristics of the UTSDC process for 3D microstructured surfaces, a set of microlens arrays was fabricated on a Moore Nanotech 350FG(Moore Nanotechnology Systems, LLC, Keene, NH, USA), as shown in Figure 3. The workpiece material is aluminum 6061-T6(Alcoa Corporation, Alcoa, TN, USA). Table 1 shows the designed parameters of the microlens arrays and the process parameters in the experiment. The diamond tool has a round nose of 0.32 mm. The tool edge radius of the diamond tool is around 0.2 μm. Four lines of microlens arrays were cut with different cutting velocities. The cutting forces were measured by a Kistler 9256C1 force transducer (Kistler Group, Winterthur, Switzerland). The sampling rate was set to 50 kHz. The surface topography was measured using a Zygo Nexview™ 3D Optical Surface Profiler (Zygo Corporation, Middlefield, CT, USA). 

Microlens arrays were cut utilizing the servo motion of the *y*- and *z*-axes of the machine tool. The corresponding tool path was determined as follows. The tool trajectory of the turning microlens arrays is shown in Figure 3b. The tool path $\left(y_{n,\;i},z_{n,\;i}\right)$ was determined by a set of cutting points denoted by ($y_{i},z_{i}$) and the increment of $y_{i}$ was set at ∆y mm. It is assumed that the length of a compound lens array is l, the pitch is p and the radius of each lens unit is the same as the tool radius, r, while the center of the *j*-th eyelet ($y_{cj},z_{cj})$ in the machine tool coordinate is expressed as:

(25) $$y_{cj}=j\times p;\;j=1,2,3\ldots ,\;\frac{l}{p}$$

In each eyelet, there are $\frac{p}{∆y}$ sampling points, and the cutting points ($y_{i},z_{i}$) for the *j*-th microlens are derived as:

(26) $$(y_{i},z_{i})=\left(y_{k+j\times p/∆y},\;\sqrt{r^{2}-{\left(y_{cj}-\left(y_{k+j\times p/∆y}\right)\right)}^{2}}\right)\;k=1,2,3,\ldots ,\;\frac{p}{∆y}$$

The tool path $\left(y_{n,\;i},z_{n,\;i}\right)$ can be derived from cutting point positions:

(27) $$(y_{n,\;i},z_{n,\;i})=(y_{i},r-s-h_{0}-z_{i})\;$$

where s is the sag value of the eyelet, such that:

(28) $$s=r-\sqrt{r^{2}-{\left(r-\left(\frac{p}{2}\right)\right)}^{2}}$$

and $y_{k+j\times p/∆y}$ satisfies Equation (29):

(29) $$y_{k+j\times p/∆y}=k\times ∆y+j\times p;\;k=1,2,3,\ldots ,\;\frac{p}{∆y}$$

In the cutting experiments, the actual uncut chip thickness is the summation of $z_{i}$ and the constant depth, $h_{0}$. $h_{0}$ is set to 5 μm, which is more than ten times larger than the tool edge radius. As a result, the tool edge effect can be ignored during the machining.

## 4. Results and Discussion

### 4.1. Surface Profile Analysis

**Figure 4:**
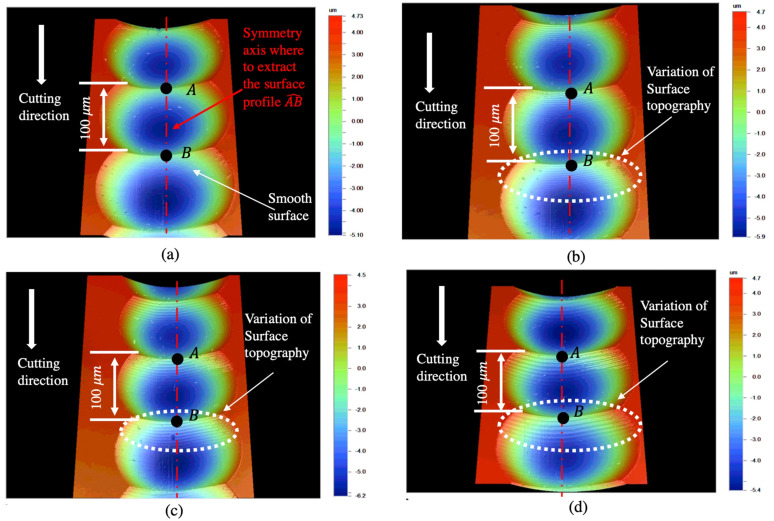
Measured surfaces: (**a**) 5 mm/min, (**b**) 10 mm/min, (**c**) 15 mm/min, (**d**) 20 mm/min.

The measured surfaces are shown in Figure 4. The surface profile $\hat{AB}$ is extracted from the surface along the axis of symmetry. Hence, the surface primary profile is obtained by removing the form of each microlens. 

**Figure 5:**
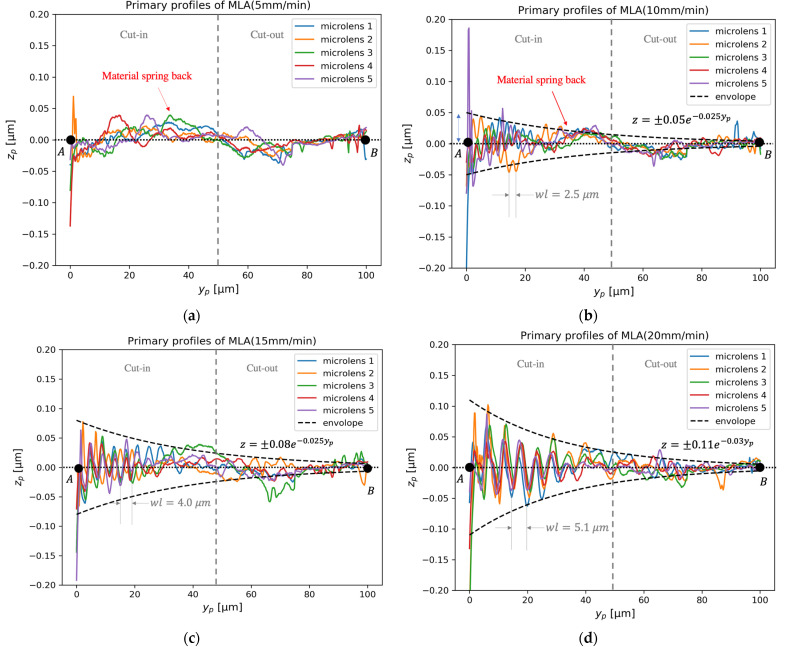
Primary profiles of microlens array corresponding to nominal cutting velocity: (**a**) 5 mm/min, (**b**) 10 mm/min, (**c**) 15 mm/min, (**d**) 20 mm/min.

The surface primary profiles corresponding to different cutting conditions are shown in Figure 5. The variation of surface primary profiles is not obvious in the profile corresponding to the relatively low cutting velocity of 5 mm/min. However, periodic components can be clearly seen in the profiles when the cutting velocity is equal to or above 10 mm/min. The wavelength (wl) of the surface topography variation can be roughly estimated by measuring the peak-to-peak distances, which are 2.5, 4.0 and 5.1 μm, as shown in Figure 5b–d, respectively. By dividing the cutting speed by the corresponding wavelength, the frequencies of the relative vibration were found to be around 60 Hz, regardless of the cutting speed. As a result, the frequency around 60 Hz was identified as the characteristic frequency of the relative tool-work vibration. The theoretical peak-to-peak distance corresponding to the characteristic frequency was around 1.4 μm when the cutting velocity was 5 mm/min. Considering that the lateral resolution of the surface profiler is around 0.2 μm, the profiler would have captured the surface variation if the relative tool-work vibration was noticeable. As shown in Figure 5c,d, the periodic components exhibit apparent damping behavior. Hence, the tool-work vibration should not be modeled as a simple undamped harmonic vibration in UTSDC. Apart from the periodic components, the material spring-back can be seen in Figure 5a,b. This coincides with Reference [19], which showed that spring-back is significant when the cutting speed is low and it contributes to the increase of the thrust force.

The DDS analysis of the surface profiles under different cutting conditions is shown in Table 2. ARMA models of order (4,3) to (12,11) are fitted to the profiles. When the cutting speed is 5 mm/min, the characteristic frequency component only contributes less than 13% of the total power of the primary profile. A pair of real roots were detected, whose percentage contribution to total power was more than 60%. The real roots represent strong break frequency with a high damping ratio (ξ>0.7). This is similar to the ultra-precision face-turning operation, where the contribution of the break frequency components was found to be above 67% of the total surface roughness [20]. As a result, the effect of relative tool-work vibration is considered to be insignificant in UTSDC when the cutting speed is less than 5 mm/min. 

When the cutting speed increased from 10 to 20 mm/min, the characteristic frequencies were found to be around 62 Hz and the damping ratio, ξ, was about 0.04. The frequencies and damping ratios appear to be independent when the cutting velocity varies in the range of 10 to 20 mm/min. However, the percentage contributions to total power were 25.97%, 79.63% and 99.12%, when the cutting speed was increased from 10 to 20 mm/min. This indicates that the relative tool-work vibration is significantly enhanced when the cutting speed increases. 

### 4.2. Estimation of the Thrust Force

**Figure 6:**
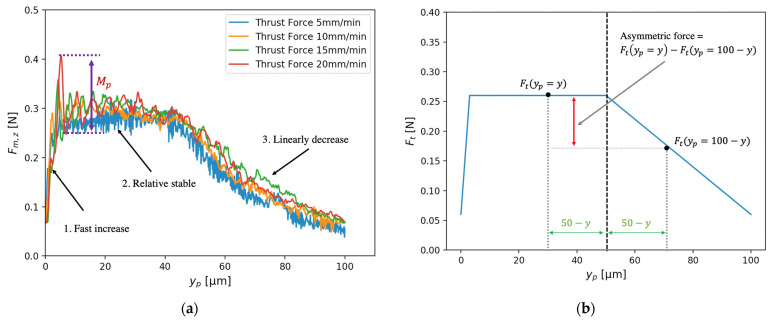
(**a**) The measured forces, $F_{m,z}$, along the thrust direction. (**b**) The thrust force, $F_{t}$, used in the dynamic model.

Figure 6 shows the measured thrust forces, $F_{m,z}$, along the thrust direction. Under the condition that the cutting speed was 5 mm/min, $F_{m,z}$ jumped from 0.06 to 0.27 N at the beginning of the cut-in process. At this stage, a certain amount of work material is indented and generates an extra resistance force on the clearance face of the diamond tool. $F_{m,z}$ then remains at a relatively stable value until the end of the cut-in process, suggesting that the volume of the indented material becomes stable. In the cut-out process, $F_{m,z}$ decreased almost linearly with increasing cutting distance. Since the vibration component is not obvious when the cutting speed is low, the spring force and damping force can be ignored. As a result, the thrust force, $F_{t}$, can be modeled by $F_{m,z}$ when v=5 mm/min,

(30) $$F_{t}\left(y_{p}\right)=F_{m,z}\left(y_{p},v=5\right)=\left\{\begin{array}{ll} 70y_{p} & y_{p}\in \left[0,\;0.003\right)\\ 0.27 & y_{p}\in \left[0.003,\;0.05\right)\;\\ 0.48-4.2y_{p} & y_{p}\in \left[0.05,\;0.1\right] \end{array}\right.$$

For an arbitrary speed v,

(31) $$y_{p}=\frac{vt}{60}$$

By combining Equations (30) and (31), the thrust force is derived as a function of time t. When v is larger, $F_{m,z}$ exhibits overshoot at the beginning of the cut-in process and the dynamic component decays as the diamond tool moves forward in the cutting direction. Although a larger cutting speed results in larger overshoot, the primary shape of $F_{m,z}$ is the same despite different cutting speeds. The frequency of the measured force, $F_{m,z}$, was around 60 Hz, which is close to the characteristic frequency identified from the surface primary profile. Since the force sampling frequency is much larger than 60 Hz, the force signal can also reflect the amplitude of the tool-work vibration.

### 4.3. Verification of the Proposed Dynamic Cutting Model

The dynamic equation of the cutting system can be derived after the $w_{n}$, ζ and $F_{t}$ are obtained from the surface profile data and thrust force data. Due to the complexity of the second-order differential equation, the numerical solution is computed using the Python scipy.integrate package. 

**Figure 7:**
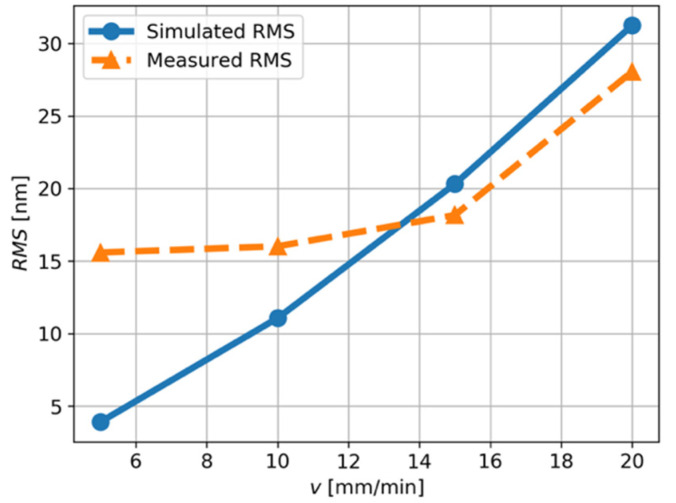
The relationship between the cutting velocity, v, and root-mean-squares $\left(\mathrm{RMS}\right)$ of the primary profile.

The root-mean-squares $\left(\mathrm{RMS}\right)$ value of the measured primary profile and the predicted profile, i.e., ${\hat{z}}_{p}$, are shown in Figure 7. The RMS values reflect the overall deviation of the machining error along the surface profile. It can be seen that the theoretical RMS value is close to the measured value when the cutting speed is 15 and 20 mm/min. Under such cutting conditions, the tool-work vibration of the diamond tool system is the major source of the surface topography variation.

When the cutting speed is smaller than 10 mm/min, the theoretical RMS value is smaller than the measured value because the vibration component only contributes to a small portion of the total power of the profile, as demonstrated in Table 2. As shown in Figure 5a,b, $z_{p}$ drops in the center of a microlens, where the cutting state changes from cut-in to cut-out. Therefore, the major error source is probably the material spring-back.

**Figure 8:**
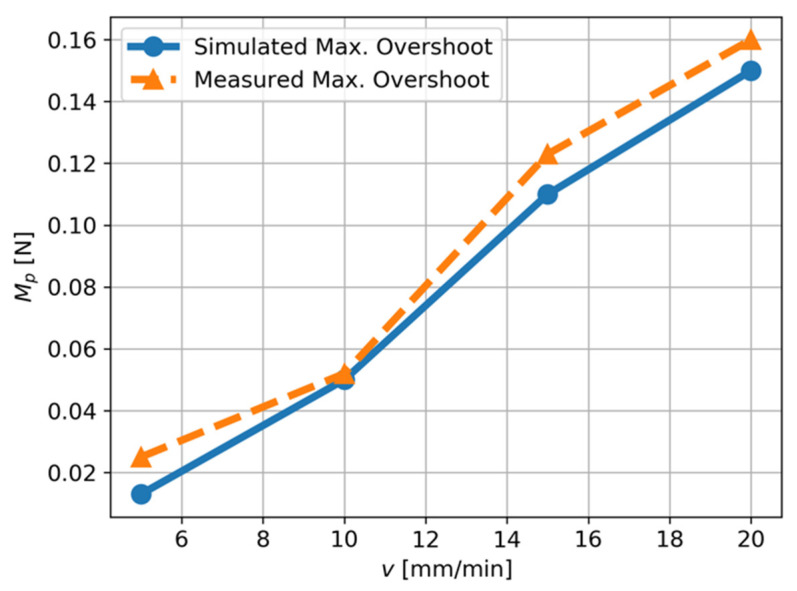
The relationship between the cutting velocity, v, and the maximum overshoot of the thrust force.

In the cut-in state, part of the material beneath the cutting tool tip is compressed and elastically recovered at the clearance face of the tool, generating an extra indentation force in the thrust direction. The extra force can be roughly measured by the asymmetric force illustrated in Figure 6b. In the cut-out process, the motion of the diamond tool is in the positive *z*-axis direction, which pushes the majority of material ahead of the tool’s rake face to the free surface. Hence, a small amount of material flows under the tool tip and the thrust force decreases. The resultant force acting on the force sensor, $F_{m,z}\left(t\right)$, can be predicted by the proposed dynamic cutting model based on Equation (21) once m, c, k, tool path trajectory and the theoretical thrust force, $F\left(t\right)$, are known. Figure 8 shows the good agreement between the maximum overshoot, $M_{p}$, of the theoretical force acting on the force sensor and the actual measured force.

Overall, the dynamic model provides insight into the formation of the surface topography variation in the UTSDC of 3D microstructured surfaces. The slide motion in the thrust direction and the sudden increase of the thrust force at the beginning of the cut-in process leads to a disturbance in the dynamic cutting system, generates an underdamped vibration of the diamond tool in the thrust direction and leaves a wavy profile in the machined surface. The effect of the cutting speed on the RMS of the surface primary profile and the maximum overshoot of thrust force are quantified based on the proposed dynamic cutting model. To strike a balance between a fine surface finish and machining efficiency, the velocity of 15 mm/min is preferred to fabricate the microlens arrays. Although the proposed method is limited by the lateral resolution of the surface profiler, and the thrust force, $F_{t}$, used as an input for the dynamic model needs to be obtained from experiments, the theoretical analysis and experimental results provide potential benefits in predicting the dynamic forces and surface topography variation in the UTSDC of microstructured surfaces. Further work is needed to calculate $F_{t}$ based on the theory of metal shearing and the tool indentation effect. The proposed dynamic model can be used in the self-optimization machining system [21], providing essential information on the process–machine interactions and machining quality inspection.

## 5. Conclusions

In this paper, the characteristics of the surface topography variation in ultra-precision tool servo-based diamond cutting (UTSDC) of 3D microstructured surfaces were investigated. Conclusions are drawn as follows:

(1) In UTSDC, the relative tool-work vibration exhibited apparent damping behavior. The vibration cannot be simplified as a steady harmonic vibration with a constant amplitude and frequency.
(2) The characteristic frequency and the damping ratio of the surface topography variation were obtained based on the data-dependent systems approach. The percentage contribution of characteristic frequency to total power of the surface topography variation increased from 12.63% to 97.98% when the cutting speed increased from 5 to 20 mm/min.
(3) Based on the analysis of the machined surface profile and thrust force, it was found that the surface topography variation in UTSDC was mainly affected by the tool-work vibration when the cutting speed was over 10 mm/min, and the variation increased with an increase of cutting speed.
(4) A dynamic model has been purposely developed which takes into account the tool path, cutting speed and the dynamics of the machining system. The model can well-predict the RMS values of the surface primary profile and the dynamic force acting on the force sensor.

## Figures and Tables

**Table 1:** The designed parameters of microlens arrays and the process parameters.

Parameter	Value
Pitch (μm)	100
Sag (μm)	3.93
Lens radius (μm)	320
Workpiece material	Aluminum 6061-T6
Cutting velocity (mm/min)	5, 10, 15, 20
Tool nose radius (μm)	320
Nominal rake angle (°)	0
Nominal clearance angle (°)	10
Constant depth of cut (μm)	5
Coolant	No

**Table 2:** Results of data-dependent systems (DDS) analysis for the tool-work vibration component along the surface primary profile.

Cutting Velocity (mm/min)	Characteristic Roots	Characteristic Frequency (Hz)	Damping Ratio	Percentage Contribution to Total Power
5	0.6139±0.8486j	59.84	0.049	12.63%
10	0.9032±0.4712j	61.39	0.039	25.97%
15	0.9560±0.3333j	63.88	0.037	79.63%
20	0.9819±0.2396j	60.03	0.045	97.98%

## Nomenclature

$\left(y_{p},z_{p}\right)$	Surface primary profile
$\left(y_{m},z_{m}\right)$	Measured profile
$\left(y_{n},z_{n}\right)$	Nominal profile/Tool path
($y_{i},z_{i}$)	Cutting location points
t	Time
$\alpha_{i}$	Autoregressive (AR) parameter
$b_{i}$	Moving average (MA) parameter
∆	Sampling interval
$\lambda_{i}$,$\;\lambda_{i}^{*}$	A pair of complex roots
$\omega_{i}$	Natural frequency
$\zeta_{i}$	Damping ratio
P	The percentage contribution of the total power
$h_{0}$	The constant depth
$z_{1}$	The displacement of the moving part of the slide along the *z*-axis
$z_{2}$	The displacement of the diamond tool along the *z*-axis
$y_{1}$	The displacement of the workpiece of the slide along the *y*-axis
$F_{m,z}$	The measured force along the *z*-axis
$F_{t}$	The thrust force
